# Inhibitor development in nonsevere hemophilia: data from the European Haemophilia Safety Surveillance (EUHASS) registry

**DOI:** 10.1016/j.rpth.2025.102887

**Published:** 2025-05-17

**Authors:** Kathelijn Fischer, Riitta Lassila, Flora Peyvandi, Alexander Gatt, Samantha C. Gouw, Robert Hollingsworth, Thierry Lambert, Radek Kaczmarek, Diana Carbonero Alvarez, Michael Makris, EUHASS participants

**Affiliations:** 1 Center for Benign Haematology, Thrombosis and Haemostasis, Van Creveldkliniek, University Medical Center Utrecht, University Utrecht, the Netherlands; 2 PedNet Haemophilia Research Foundation, Baarn, the Netherlands; 3 Department of Hematology, Unit of Coagulation Disorders, Helsinki University Central Hospital, Research Program Unit in Systems Oncology, University of Helsinki, Helsinki, Finland; 4 Angelo Bianchi Bonomi, Hemophilia and Thrombosis Centre, Fondazione IRCCS Ca’ Granda Ospedale Maggiore Policlinico; 5 Department of pathophysiology and Transplantation, University of Milan, Milan, Italy; 6 Mater Dei Hospital, Tal-QRoqq, Msida, Malta; 7 Amsterdam UMC location University of Amsterdam, Amsterdam, Netherlands; 8 Department of Pediatric Hematology, Meibergdreef 9, Amsterdam, the Netherlands; 9 Medical Data Solutions and Services (MDSAS), Manchester, UK; 10 Reference Center for hemophilia and rare bleeding disorders, Hôpital Bicêtre, APHP, Université Paris Saclay. Le Kremlin Bicêtre, France; 11 Coagulation Products Safety Supply and Access Committee, World Federation of Hemophilia, Montreal, Quebec, Canada; 12 Wells Center for pediatric Research, Indiana University School of Medicine, Indianapolis, USA; 13 European Association of Haemophilia and Associated Disorders, Brussels, Belgium; 14 School of Medicine and Population Health, University of Sheffield, Sheffield, UK

**Keywords:** inhibitor, factor VIII, factor IX, hemophilia, antibodies, neutralizing

## Abstract

**Background:**

Information on inhibitor development in nonsevere hemophilia and its association with clotting factor concentrate type is limited.

**Objectives:**

To assess inhibitor development in patients with nonsevere hemophilia A (HA) and hemophilia B (HB) in the European Haemophilia Safety Surveillance system.

**Methods:**

Inhibitors and total treated patients are reported annually. Any exposure to concentrate per year was considered a treatment year. Incidence rates per 1000 treatment years and 95% CIs were calculated according to type of concentrate and compared using incidence rate ratios (IRRs).

**Results:**

During 2008 to 2023, 90 centers reported on 36,074 (HA) and 9238 (HB) treatment years. The inhibitor rate for nonsevere HA receiving factor (F)VIII was 4.2 per 1000 treatment years (95% CI, 3.5-4.9). Inhibitors developed at median 47.5 years (P25-P75 [IQR], 17.0-69.0), after median 40 exposure days (EDs; IQR, 17-80), with 58% occurring <50 EDs and 88% <100 EDs. Overall, 4 of 149 (2.7%) patients in the inhibitor group were female. Only one inhibitor was reported in nonsevere HB, in a female patient (FIX 7%, after 6 EDs), resulting in an inhibitor rate of 0.1 per 1000 treatment years (95% CI, 0.0-0.6). Compared with standard half-life recombinant FVIII, inhibitor rates on both plasma-derived FVIII (IRR, 0.27; 95% CI, 0.11-0.58; *P* < .001) and extended half-life FVIII (IRR, 0.18; 95% CI, 0.02-0.68; *P* = .002) were significantly reduced.

**Conclusion:**

Inhibitors in nonsevere hemophilia occurred at a rate of 4.2 per 1000 treatment years in HA and 0.1 per 1000 treatment years in HB. Compared with standard half-life FVIII, inhibitor development on plasma-derived and extended half-life FVIII were reduced. These data show that inhibitor monitoring is relevant with nonsevere HA in both sexes and should be continued lifelong.

## Essentials

• Inhibitor formation in nonsevere hemophilia A (36,074 years) and B (9238 years) was analyzed.
• Inhibitor rates were 4.2 per 1000 in hemophilia A and 0.1 per 1000 treatment years in hemophilia B.
• Inhibitor development was lower with plasma-derived and extended half-life FVIII concentrates.
• Lifelong inhibitor monitoring is relevant in both men and women with nonsevere hemophilia.

## 1 Introduction

Inhibitory antibodies (inhibitors) against exogenous factor (F)VIII or IX concentrates interfere with their therapeutic potential in patients with hemophilia by blocking correction of FVIII/FIX-driven coagulation in case of treatment of bleeds or surgeries as well as effective prophylactic replacement therapy in patients with hemophilia. Large studies have shown that inhibitors occur most frequently in previously untreated patients (PUPs) during the first 50 exposure days (EDs) in severe hemophilia A (±30% cumulative incidence) and hemophilia B (±10% cumulative incidence), respectively [1,2]. After the first 50 EDs, inhibitors still occur, albeit much less frequently [3].

For nonsevere hemophilia A and B, however, the inhibitor incidence is less well established. Cumulative incidence can only be established by following individual patients, which requires extended periods for patients who are treated infrequently, such as those with nonsevere hemophilia. The largest data on cumulative incidence of inhibitors in nonsevere hemophilia A were from the international study on etiology of inhibitors in patients with moderate or mild hemophiolia A (INSIGHT), which followed 1112 patients with nonsevere hemophilia from 14 treatment centers for 30 years and reported a cumulative incidence of 6.7% (95% CI, 4.5%-8.9%) at 50 EDs, increasing up to 13.3% (95% CI, 9.6%-17.0%) after 100 EDs [4].

Registries collecting data anonymously can only report on incidence rates: ie, the number of patients developing inhibitors divided by the number of patients treated with clotting factor concentrates in that year. Since 2008, the European Haemophilia Safety Surveillance (EUHASS) registry has collected data on treatment in severe and nonsevere hemophilia and other side effects according to clotting factor concentrate [5]. In 2015, it reported rates of inhibitor development in 7969 patients with nonsevere hemophilia A (inhibitor rate of 4.3/1000 treatment years; 95% CI, 3.0–5.9) and 1863 patients with nonsevere hemophilia B (inhibitor rate of 0.5/1000 years; 95% CI, 0.01–2.6) from 68 treatment centers during 4 years [6].

More information on inhibitor development in nonsevere hemophilia for a longer period of follow-up may be used for counseling of patients and may serve as a model for patients with severe hemophilia on prophylaxis with nonreplacement therapy, who will receive infrequent treatment with factor concentrates only.

The present data are an extension of the earlier EUHASS report on inhibitor development in nonsevere hemophilia from 2008 until 2012 [6], reporting on prospectively collected data on inhibitor development according to FVIII/IX concentrates from 90 centers collected during 14.25 years (2008-2023).

## 2 Methods

This analysis includes data collected during 14.25 years from October 1, 2008, to January 1, 2023, from 95 European hemophilia treatment centers participating in the EUHASS registry. A list of participating centers is provided in the Supplementary Material. The design of the EUHASS registry has been described previously [5,7]. Briefly, for each clotting factor concentrate, the total number of patients, and the number of patients with severe hemophilia treated were reported annually for each participating center. The number of treatment years for patients with nonsevere hemophilia (endogenous FVIII/IX activity, 0.01-0.40 IU/mL or 1%-40%) was established indirectly by subtracting the number of treated patients with severe hemophilia (endogenous FVIII/IX activity, <0.01 IU/mL or <1%) from the total number of treated patients. Consequently, a treatment year was defined by any exposure to a clotting factor concentrate in that year and independent of the number of EDs to that concentrate. Concomitantly, the occurrence of new inhibitors was reported quarterly, including diagnosis, endogenous FVIII/IX activity level, age, number of EDs before inhibitor development, and concentrate used before inhibitor development. Inhibitor testing was performed according to local protocol in local laboratories; inhibitors were defined by 2 positive tests and expressed in Bethesda Units (BU). Recurrent inhibitors were excluded from analysis. For inhibitor patients, EDs were recorded up to 1000 EDs and coded as >999 EDs for patients with 1000 EDs or more. For noninhibitor patients, data were only collected at group level, divided by severe and nonsevere hemophilia, to ensure anonymity and only information regarding the concentrate received was collected annually, while information on the number of EDs or any other patient characteristics was unavailable. Only data on years with both information on the number of patients treated and inhibitor development were included in the analyses.

Before study entry, all centers obtained approval from their institutional review boards. Individual informed consent was not obtained as all data were collected at group level, and inhibitor data were coded.

### 2.1 Statistical analysis

Data were analyzed separately for patients with hemophilia A and B. As some of the characteristics for inhibitor patients had a skewed distribution, descriptive statistics were presented as medians and interquartile ranges (IQR; P25-P75). Inhibitor development was expressed as incidence rates per 1000 treatment years (incidence rate ratio [IRR] with 95% CIs).

For patients with hemophilia A, characteristics of inhibitor patients according to endogenous FVIII activity levels (1%-5%, 6%-10%, and 11%-40% respectively; with an additional comparison of 1-2% vs 3-5%), and age at inhibitor development (up to 18 vs 19-49 vs 50-69 vs ≥70 years) were compared. Inhibitor development rates according to concentrate type (plasma-derived [pd] concentrates [pdFVIII], standard half-life [SHL] recombinant concentrates [SHL-FVIII], and extended half-life [EHL] recombinant concentrates [EHL-FVIII]) were compared. Patients on nonreplacement therapy (for hemophilia A only, emicizumab) or unlicensed study drugs were excluded from the analysis. In addition, data on patients treated with FVIII concentrates with high von Willebrand factor content (Fanhdi, Haemate P, Voncento, Wilate, Wilfactin, and Wilstart) were excluded, as these most likely represent and/or include patients treated for von Willebrand disease. Inhibitor development according to individual concentrates was compared only for FVIII/IX concentrates with a minimum of 1000 treatment years.

For hemophilia B, the low reported number of inhibitors precluded analyses of individual concentrates. The 95% CIs were calculated using the exact method [8]. Characteristics of inhibitor patients across endogenous FVIII activities and across age at inhibitor development were compared using Kruskal–Wallis tests for continuous variables and chi-squared tests for continuous variables. Inhibitor rates were compared using IRR with their CI. IRR were calculated using Medcalc [9]. Descriptive statistics were performed using SPSS 29.0 (IBM).

## 3 Results

During the observation period of 14.25 years, 90 of 95 (95%) hemophilia treatment centers from 28 European countries participating in EUHASS contributed data on both inhibitor development and the number of treated patients with nonsevere hemophilia.

The number of patients with nonsevere hemophilia registered varied slightly per year. In 2022, EUHASS collected data on 9614 patients with nonsevere hemophilia A and 2274 patients with nonsevere hemophilia B. Overall, the centers reported 36,074 treatment years for nonsevere hemophilia A and 9238 treatment years for nonsevere hemophilia B. Treatment years (*n* = 259) on emicizumab (Hemlibra) were excluded from analysis, as well as 73 treatment years (without inhibitors) on unlicensed clinical products, which included both FVIII and FIX concentrates. A total of 154 cases of FVIII inhibitor development were reported, of which 5 (3%) were excluded due to failure to report the number of patients treated in that center for that year. The inhibitor rate for nonsevere hemophilia A was 4.2 per 1000 treatment years (95% CI, 3.5-4.9). Only 1 FIX inhibitor was reported in nonsevere hemophilia B, resulting in an inhibitor rate of 0.1 per 1000 treatment years (95% CI, 0.0-0.6). During the period of 14 years, these 155 inhibitors represented 25.5% (155/608) of all FVIII/IX inhibitors reported to EUHASS, the other inhibitors were reported in patients with severe hemophilia and were presented in previous analyses and the EUHASS annual reports from 2019 to 2022 [3,10].

### 3.1 Characteristics of inhibitor patients

Characteristics of patients developing inhibitors are shown in Table 1. In patients with hemophilia A, 42% of inhibitors were observed in patients with moderate hemophilia (FVIII activity, 1%-5%) and 25% in patients with FVIII activities between 6% and 10%, and 33% in those with higher FVIII activities (Figure). Of note, only 3 of 149 inhibitor patients (2%) had an endogenous FVIII activity of >25%.

**Table 1:** Characteristics of inhibitor patients in nonsevere hemophilia according to diagnosis.

Characteristic	Hemophilia A	Hemophilia B
	Median (IQR) or *n* (%)	
No.	149	1
Age (y)	47.5 (17.0-69.0); range, 0.7-86.0	26
Female sex	4 (3)	1 (100)
Moderate hemophilia	62 (42)	0
Endogenous FVIII/FIX level (%)	7 (3-15)	7
No. of EDs before inhibitor development	40 (16-80); range, 3-750	6
Inhibitors within the first 50 EDs	86 (58)	1 (100)
Median first titer (BU)	2.3 (1.0-6.0); range, 0.3-55	5.0
Percentage of high titer within first 2 measurements	53 (36)	0

BU, bethesda units; ED, exposure day; F, factor.

**Figure:**
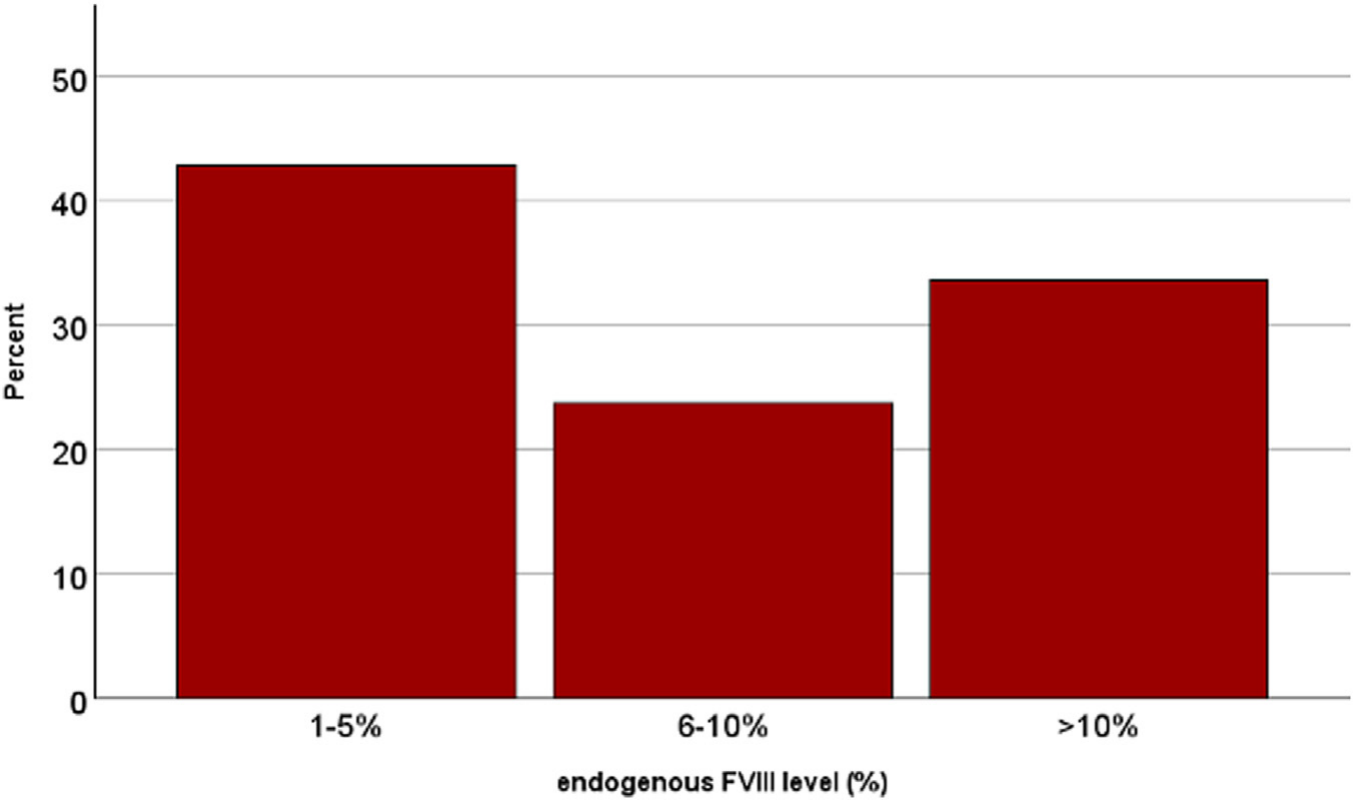
Distribution of inhibitors according to endogenous factor VIII activity.

Patients developed FVIII inhibitors at a median age of 47.5 years (IQR, 17.0-69.0), after a median of 40 EDs (IQR, 16-80); 58% of inhibitors occurred before reaching 50 EDs, 30% between 50 and 100 EDs, and 12% occurred after 100 EDs. The median first inhibitor titer was 2.3 BU/mL (IQR, 1.0-6.0), including 36% with a high-titer inhibitor (>5 BU/mL). Interestingly, 4 of 149 (2.7%) inhibitor patients were female, developing inhibitors at a median age of 29.4 years after a median of 20 EDs. As details on noninhibitor patients were not collected in the EUHASS registry, inhibitor rates according to sex or FVIII activity levels could not be calculated.

One inhibitor was observed in a female patient with mild hemophilia B (FIX activity, 7%); this inhibitor developed at age 26 years, after 6 EDs, with a first inhibitor titer of 5.0 BU and a second titer of 2.0 BU. No male individuals with nonsevere hemophilia B developed inhibitors.

### 3.2 Inhibitor characteristics according to endogenous FVIII levels

As treatment patterns are likely to vary across endogenous FVIII activities and may affect inhibitor risk, inhibitor characteristics were compared according to categories of endogenous FVIII (Table 2). This analysis showed that these were similar across the 3 groups. Median age at inhibitor development varied between 40.7 and 52.1 years (*P* = .349), and median inhibitor titers varied between 2.0 and 2.8 BU (*P* = .50), including around one-third with high titers. The most striking finding was that inhibitors in patients with moderate hemophilia A developed after significantly more EDs: median 75 EDs compared with 27 EDs in those with 6% to 10% endogenous FVIII activity and 25 EDs in those with 11% to 40% endogenous FVIII activity (*P* < .001). As it is well known that patients with FVIII levels of 1% to 2% have a more severe phenotype, we performed an additional analysis comparing patients with 1% to 2% FVIII activity (*n* = 29) and with 3% to 5% FVIII activity (*n* = 34). This analysis did not show clear differences between these 2 subgroups of severity: although median age at inhibitor development was earlier in the group with 1% to 2% of FVIII activity at a median age of 30.3 years (IQR, 7.9-69.9) vs at 53.3 years (IQR, 11.1-66.4) in those with 3% to 5% FVIII activity, the interquartile ranges still overlapped and the statistical comparison was nonsignificant (*P* = .39). The median number of EDs was similar at 75 (IQR, 22-106) and 65 EDs (IQR, 40-100) respectively, resulting in a *P* value of .93.

**Table 2:** Characteristics of patients with nonsevere hemophilia and FVIII inhibitors according to endogenous FVIII activity.

Characteristic	Moderate (1%-5%)	Mild (6%-10%)	Mild (11%-40%)	*P*
	Median (IQR) or *n* (%)			
No.^a^	63 (42.2)	36 (24.2)	50 (33.6)^a^	
Age at inhibitor development (y)	40.7 (10.4-65.3); range, 0.7-79.0	52.1 (26.7-67.0); range, 1.2-86.0	48.9 (18.5-70.5); range, 1.8-85.3	.349
Female sex	2 (3.2)	0	2 (4.0)	.50
No. of EDs before inhibitor development	75 (31-100); range, 3-750	27 (15-59); range, 4-100	25 (14-70); range, 6-200	<.001
Inhibitors within the first 50 EDs	27 (42.9)	24 (66.7)	35 (70.0)	.007
Median first titer (BU)	2.8 (1.0-7.4); range, 0.4-55	2.0 (0.8-4.1); range, 0.3-45.5	2.4 (1.0-4.9); range, 0.5-30	.50
Percentage of high titer within first 2 measurements	24 (38.1)	10 (27.8)	19 (38.8)	.51

BU, bethesda units; ED, exposure day; F, factor.

a Including 3 patients with FVIII activities >25%.

### 3.3 Inhibitor characteristics according to age at inhibitor diagnosis

Based on the report of a bimodal distribution of inhibitor development from the United Kingdom, children aged up to 18 years were divided into 3 groups. However, no clear trend in children could be observed: 9% of inhibitors occurred <6 years, 7% between 6 and 9 years, and 13% between 10 and 18 years. As the number of patients was low, it was decided to perform the analyses in 3 global age groups: up to age 18 years (*n* = 43, 28.9%), 19 to 49 years (*n* = 35, 23.3%), 50 to 69 years (*n* = 38, 25.5%), and 70 years and older (*n* = 33, 22.1%). Results of this analysis are shown in Table 3. It shows similar endogenous FVIII activities (median, 5%- 7%), a nonsignificant downward trend in the proportion of patients with moderate hemophilia with increasing age (from 53.5% to 8.7%; *P* = .52) and a similar number of EDs to FVIII before inhibitor development (median, 34-45 EDs). Inhibitors developed somewhat earlier in the youngest group with 72.1% developing within the first 50 EDs compared with approximately 50% in the older groups (*P* = .14). First inhibitor titers were similar, but we noted a significantly increased proportion of high-titer inhibitors in the youngest group at 58.1%, compared with 20.0% to 34.2% in the older groups (*P* = .002).

**Table 3:** Characteristics of patients with nonsevere hemophilia and FVIII inhibitors according to age at inhibitor development.

Characteristic	Up to 18 y	19-49 y	50-69 y	≥70 y	*P*
	Median (IQR) or *n* (%)				
No.	43 (28.9)	35 (23.5)	38 (25.5)	33 (22.1)	
Endogenous FVIII activity (%)	5 (2-12); range, 1-20	7 (3-16); range, 1-27)	7 (5-15); range, 1-39)	7 (3-13); range, 1-24)	.353
Moderate hemophilia	23 (53.5)	12 (34.3)	15 (10.1)	13 (8.7)	.523
Female sex	2 (4.7)	1 (2.9)	0	1 (3.0)	.636
No. of EDs before inhibitor development	37 (14-63); range, 3-113	34 (19-99); range, 4-250	45 (199-80); range, 6-750	43 (15-120); range, 4-350	.453
Inhibitors within the first 50 EDs	31 (72.1)	18 (51.4)	21 (55.3)	16 (48.5)	.141
Median first titer (BU)	4.0 (1.0-10.0); range, 0.3-55.0	1.9 (1.0-4.7); range, 0.5-22.9	2.2 (1.0-6.3); range, 0.4-45.5	2.1 (0.8-4.2); range, 0.5-16.0	.159
Percentage of high titer within first 2 measurements	25 (58.1)	7 (20.0)	13 (34.2)	8 (24.2)	.002

BU, bethesda units; ED, exposure day; F, factor.

### 3.5 Inhibitor development according to concentrate type

Inhibitor development according to types of FVIII and FIX concentrates is shown in Table 4. The number of treatment years on EHL-FVIII (*n* = 2,200) was much lower than those for the SHL-FVIII concentrates (*n* = 28,395). Notwithstanding, the inhibitor rate on EHL-FVIII was significantly lower, with an IRR of 0.18 (95% CI, 0.02-0.68; *P* = .002). In addition, inhibitor development in patients treated with pdFVIII was also significantly lower compared with SHL-FVIII, at an IRR of 0.27 (95% CI, 0.11-0.58; *P* < .001). The group of SHL-FVIII concentrates included 9 concentrates, of which 4 had >1000 treatment years and for which inhibitor development was compared separately (Supplementary Table 1). In this analysis, only Refacto AF showed a significantly higher inhibitor rate (IRR, 2.72; 95% CI, 1.74-4.27; *P* < .001) than Advate.

**Table 4:** Inhibitor development in nonsevere hemophilia according to concentrate type.

Treatment	No. inhibitor	No. treatment years	Inhibitor rate/1000 y	95% CI	Unadjusted incidence rate ratio: IRR (95% CI); *P*
Hemophilia A					
All treated with FVIII	149	35,815	4.2	3.5-4.9	
SHL-FVIII	140	28,395	4.9	4.1-5.8	Reference
EHL-FVIII	2	2200	0.9	0.1-3.3	0.18 (0.02-0.68); .002
pdFVIII	7	5220	1.3	0.5-2.8	0.27 (0.11-0.58); <.001
Hemophilia B					
All treated with FIX	1	9238	0.1	0.0-0.6	
SHL-FIX	1	5938	0.2	0.0-0.9	Reference
EHL-FIX	0	1134	0.0	0.0-3.2	0.00 (0.00-204.22); NS
pdFIX	0	2166	0.0	0.0-1.7	0.00 (0.00-106.92); NS

EHL, extended half-life; F, factor; NS, not significant; pd, plasma-derived; SHL, standard half-life.

The group of EHL-FVIII concentrates included 4 concentrates, of which 62.8% of treatment years were represented by Elocta, which showed only a statistically nonsignificant trend toward reduced inhibitor development compared with Advate (IRR, 0.41; 95% CI, 0.05-1.60; *P* = .207).

The group of pdFVIII showed significantly reduced inhibitor development, but it included as many as 13 individual concentrates. Of these, only Emoclot (*n* = 1019) had more than 1000 treatment years, showing a nonsignificant trend toward reduced inhibitor development compared with Advate (IRR, 0.00; 95% CI, <0.01 to 1.09).

For FIX concentrates, only 1 inhibitor was observed, and the number of treatment years was much lower, which hampered reliable calculations of incidence rate ratios among SHL-FIX, EHL-FIX, and pdFIX (Table 4). Details on the individual SHL-FIX (*n* = 2), EHL-FIX (*n* = 3), and pdFIX (*n* = 13) are shown in supplemental data (Supplementary Table 2).

## 4 Discussion

The presented data, prospectively collected from 90 hemophilia centers during 14 years, to our knowledge, represent the largest data on inhibitor development in nonsevere hemophilia to date. In this period, 25% of all FVIII/IX inhibitors occurred in nonsevere hemophilia.

In nonsevere hemophilia A, 149 inhibitors developed in 35,815 FVIII treatment years, resulting in an inhibitor rate of 4.2 per 1000 treatment years (95% CI, 3.5-4.9). FVIII inhibitors developed at a median age of 47.5 years with 58% occurring before 50 EDs, and 4 of 149 (2.7%) occurring in females.

In nonsevere hemophilia B, 1 inhibitor developed in 9238 treatment years, resulting in an inhibitor rate of 0.1 per 1000 treatment years (95% CI, 0.0-0.6). This FIX inhibitor developed in a female patient after 6 EDs.

When comparing inhibitor development according to type of FVIII concentrates, the data suggested lower inhibitor development on EHL-FVIII (IRR, 0.18; 95% CI, 0.02-0.68; *P* = .002) and pdFVIII (IRR, 0.27; 95% CI, 0.11-0.58; *P* < .001) compared with that on SHL-FVIII. However, when comparing inhibitor development on individual concentrates to Advate, only Refacto AF showed significantly increased inhibitor rate (IRR, 2.72; 95% CI, 1.74-4.27; *P* < .001)

### 4.1 Comparison with other studies

The present inhibitor rates are in accordance with those observed in the analysis of the data from the first 4 years of EUHASS [6], reporting an inhibitor rate of 4.3 per 1000 treatment years (95% CI, 0.3-5.9) for nonsevere hemophilia A. For nonsevere hemophilia B, no additional inhibitors were reported, resulting in a reduction of the inhibitor rate with a narrowing of the 95% CI from 0.5 per 1000 (95% CI, 0.01–2.6) to 0.1 per 1000 treatment years (95% CI, 0.0-0.6) in this analysis. Comparison of inhibitor incidence with other reports is difficult due to differences in study design: (inter)national cohort studies usually present a cumulative incidence of inhibitor development according to EDs. For nonsevere hemophilia A, the INSIGHT study reported on 2711 patients ever exposed to FVIII from 34 centers [4]. Using survival analysis until 200 EDs, cumulative inhibitor incidence at 50 EDs was 6.7% (95% CI, 4.5-8.9), increasing to 13.3% (95% CI, 9.6-17.0) at 100 EDs. For nonsevere hemophilia B, we could not identify reports on either cumulative incidence or incidence rates of inhibitor development. Based on the American Universal Data Collection study, Puetz et al. [11] show that 64% of all patients with hemophilia B were nonsevere and report a prevalence of FIX inhibitors of 0.33% (8/2403) without reporting on FIX exposure in noninhibitor patients [11]. In an in-depth discussion of inhibitor development in hemophilia B, DiMichele [12] reports 1% to 3% cumulative FIX inhibitor incidence across all severities and suggests that the lower inhibitor incidence in hemophilia A compared with that in hemophilia B may depend on the higher proportion of patients with nonsevere hemophilia, the presence of crossreactive material, mutation type, or the structural similarity of FIX to other vitamin K–dependent clotting factors.

As information on treatment intensity in patients with nonsevere hemophilia was not collected, and it may take decades to reach 50 EDs in a patients with nonsevere hemophilia, any comparisons between nonsevere and severe hemophilia A or B must be interpreted with caution. Moreover, the number of EDs per year in severe hemophilia is expected to be consistently much higher. Without considering these differences in intensity of exposure, the presented data suggest a significantly higher inhibitor incidence than for patients with severe hemophilia A after 50 EDs (previously treated patients [PTPs]) reported by EUHASS: at 4.2 per 1000 in nonsevere versus 1.0 per 1000 treatment years in severe PTPs. In contrast, inhibitor incidence appeared lower for nonsevere hemophilia B at 0.1 vs 0.4 per 1000 treatment years in PTPs with severe hemophilia B, albeit with overlapping CIs [3].

Characteristics of FVIII inhibitor patients were similar to our first report [6]: median age at inhibitor development (47.5 vs 35.3 with overlapping IQR), proportion with moderate hemophilia (42 vs 36%), and 58% vs 72% developing within the first 50 EDs. Unfortunately, information on sex was included in this analysis only. Inhibitor characteristics were also consistent with those reported over 2000 to 2010 by the INSIGHT study, which occurred at a median age of 46 years (IQR, 18-65), with 69% developing before reaching 50 EDs [4]. The presented data did not corroborate the bimodal distribution of age at inhibitor development in patients with severe hemophilia in the United Kingdom previously reported by Hay et al. [13].

Regarding inhibitor development according to FVIII concentrates, a trend toward lower inhibitor rates on pdFVIII was already observed in the first EUHASS analysis of nonsevere hemophilia, while data on EHL concentrates were not available at that time [6]. However, a trend toward reduced inhibitor development on pdFVIII was not observed in severe PTPs (IRR, 1.01; 95% CI, 0.56-1.75) [3] nor in 442 intensively treated severe PUPs from Europe and Canada participating in both EUHASS and the PedNet registry (odds ratio [OR], 1.27; 95% CI, 0.70-2.27; *P* = .439). However, it was corroborated in the analysis of 767 PUPs who participated in EUHASS and the Canadian Safety Surveillance system (CHESS) only, showing a significantly reduced inhibitor rate on pdFVIII, with an OR of 0.45 (95% CI, 0.27-0.98; *P* = .003 [10]. The INSIGHT consortium performed a nested case–control study comparing inhibitor development in 298 patients with nonsevere hemophilia A. They observed a trend toward reduced inhibitor development on pdFVIII (OR, 0.59; 95% CI, 0.31-1.10) in their unadjusted analysis, which disappeared after adjustment for other risk factors (OR, 1.04; 95% CI, 0.40-2.78) [14]. This shift in ORs shows that inhibitor risk may depend heavily on other factors than the type of FVIII concentrate used and that unadjusted OR or IRR should be interpreted with caution.

Despite the large overall cohort, the analysis is limited by low patient numbers, especially regarding EHL-FVIII. The finding that inhibitor development was reduced in patients treated with EHL-FVIII is corroborated by EUHASS analyses in severe PTPs (IRR, 0.12; 95% CI, <0.01 to 0.70; *P* < .01) but not in PUPs with severe hemophilia where inhibitor development on EHL-FVIII was similar to that on SHL-FVIII at (22.2% vs 26.9%; OR, 0.78; 95% CI, 0.40-1.50; *P* =.45) [3,10].

### 4.2 Clinical implications

The present data show that patients with nonsevere hemophilia, including females, should be tested for inhibitor development after exposure to clotting factor concentrates. In addition, it is important to appreciate that in nonsevere hemophilia A inhibitor development risk persists. With 42% of inhibitors occurring after 50EDs, mostly well into adulthood, continued regular monitoring of inhibitor development in nonsevere hemophilia is needed.

We were unable to identify trends regarding endogenous FVIII level or age within the group of inhibitor patients. Due to the lack of details on noninhibitor patients and paucity of data, EUHASS was unable to study other risk factors than (type of) FVIII concentrates or perform comparisons adjusted for other risk factors. The role of pdFVIII and EHL-FVIII in the context of intensive treatment remains to be elucidated. The subanalysis of the data from EUHASS and CHESS in PUPs with severe hemophilia A, suggests that pdFVIII may be associated with reduced inhibitor development in those without intensive treatment in their early youth. The treatment intensity may also contribute to the conflicting conclusions on inhibitor development in severe hemophilia A PUPs of the randomized Survey of Inhibitors in Plasma-Product treated Toddlers study and the PedNet registry [15,16]. Moreover, as they need to choose one concentrate for their patients, clinicians need information on inhibitor risk of individual concentrates, and this is still very scant. When considering inhibitor risk in severe patients on prophylaxis with nonreplacement therapy who have only intermittent exposure to FVIII, the data on patients with nonsevere hemophilia could be considered as a model for the treatment-related risk factors of inhibitor development. Although the baseline inhibitor risk of patients with severe hemophilia will always exceed that of patients with nonsevere hemophilia due to the absence of endogenous FVIII/FIX, EUHASS will continue to collect data on inhibitor development according to FVIII/IX concentrates. More detailed information on risk factors for inhibitor development requires detailed data collection dependent on informed consent, such as that obtained in the various national registries.

Regarding treatment of the inhibitors, EUHASS did not collect any data. However, the majority are low-titer inhibitors, which is associated with successful eradication in severe hemophilia A, while reports in nonsevere hemophilia A are conflicting: 1 case series of 36 patients with nonsevere hemophilia A and inhibitors showed no association of initial and peak inhibitor titers with clearance of the inhibitor [17], while, in another case series, all 5 patients with nonsevere hemophilia A and inhibitor titers between 2 and 32 BU/mL were successfully tolerized with immune tolerance induction therapy [18].

### 4.3 Strengths and limitations

Inherent to the strength of the large size of this data set is the lack of details, especially regarding the characteristics of noninhibitor patients. The only data on these patients collected in the EUHASS registry are diagnosis, severity at category/group level, and concentrate used. Unfortunately, these data cannot provide inhibitor incidences according to sex or baseline clotting factor levels, nor provide additional insight on genetic and nongenetic risk factors or on the prognosis of inhibitor patients. A strength of our analysis is that only years with exposure to clotting factor concentrates (treatment years) were considered, generating information on inhibitor rates according to diagnosis and concentrate use. But, even in this data set, a comparison of many individual FVIII/IX concentrates is constrained by limited numbers, especially for recently introduced concentrates.

## 5 Conclusion

In this large study monitoring around 12,000 patients with nonsevere hemophilia for 14 years, the inhibitor rate for nonsevere hemophilia A was 4.2 per 1000 treatment years (95% CI, 3.5-4.9). FVIII inhibitors were developed at a median age of 47.5 years, after a median of 40 EDs, with 58% developing in the first 50 EDs. Overall, 3% of FVIII inhibitors occurred in females. Compared with SHL-FVIII, inhibitor development on pdFVIII and EHL-FVIII was lower, but individual concentrates with reduced inhibitor development could not be identified. For nonsevere hemophilia B, the inhibitor rate was 0.1 per 1000 treatment years (95% CI, 0.0-0.6), with one female patient developing an inhibitor at age 26 years after 6 EDs. These data support the need for monitoring of inhibitor development in both males and females with nonsevere hemophilia and should be continued well into adulthood and after 50 EDs.
