# Supplementary material for: Inhibitor development in nonsevere hemophilia: data from the European Haemophilia Safety Surveillance (EUHASS) registry
Source: Res Pract Thromb Haemost. 2025 May 17;9(4):102887. doi: 10.1016/j.rpth.2025.102887 (PMC12178916; doi:10.1016/j.rpth.2025.102887)
Supplement: Supplementary Data 1 [file mmc1.docx]

SUPPLEMENTAL DATA

Table S1 Inhibitor development according to concentrate in non-severe hemophilia A

| **Concentrate** | **Inhi-bitor**  **(N)** | **Treat-ment years**  **(N)** | **inhibitor rate/**  **1000**  **yrs** | **CI** | **Unadjusted IRR** | **IRR CI** | **p-value** |
| --- | --- | --- | --- | --- | --- | --- | --- |
| **Hemophilia A** |  |  |  |  |  |  |  |
| Advate+iblias | 38 | 10941 | 3.5 | 2.4-4.8 | REF |  |  |
| Afstyla | 1 | 255 | 3.9 | 0.1-21.7 |  |  |  |
| Kogenate/Helixate | 35 | 7442 | 4.7 | 3.3-6.5 | 1.35 | 0.83-2.20 | 0.198 |
| Kovaltry | 1 | 1263 | 0.8 | 0.0-4.4 | 0.23 | 0.01-1.35 | 0.092 |
| NovoEight | 9 | 2066 | 4.4 | 2.0-8.3 | 1.25 | 0.53-2.64 | 0.530 |
| Nuwiq+Vihuma | 2 | 803 | 2.5 | 0.3-9.0 |  |  |  |
| Recombinate | 2 | 237 | 8.4 | 1.0-30.1 |  |  |  |
| Refacto | 3 | 201 | 14.9 | 3.1-43.0 |  |  |  |
| Refacto AF | 49 | 5187 | 9.4 | 7.0-12.5 | 2.72 | 1.74-4.27 | <0.001 |
|  |  |  |  |  |  |  |  |
| Adynovi/  Adynovate | 0 | 270 | 0.0 | 0.0-13.6 |  |  |  |
| Elocta (Eloctate) | 2 | 1381 | 1.4 | 0.2-5.2 | 0.41 | 0.05-1.60 | 0.207 |
| Esperoct (N8-GP) | 0 | 394 | 0.0 | 0.0-9.3 |  |  |  |
| Jivi | 0 | 155 | 0.0 | 0.0-23.5 |  |  |  |
|  |  |  |  |  |  |  |  |
| Aafact | 2 | 141 | 14.2 | 1.7-50.3 |  |  |  |
| Alphanate | 0 | 104 | 0.0 | 0.0-34.8 |  |  |  |
| Amofil | 0 | 97 | 0.0 | 0.0-37.3 |  |  |  |
| Beriate | 0 | 802 | 0.0 | 0.0-4.6 |  |  |  |
| Emoclot/Umaclot/Klott | 0 | 1019 | 0.0 | 0.0-3.6 | 0.00 | <0.01-1.10 | NA |
| Factane (LFB) | 2 | 659 | 3.0 | 0.4-10.9 |  |  |  |
| Factor 8Y (BPL) | 0 | 158 | 0.0 | 0.0-23.1 |  |  |  |
| Faktor VIII SDH Intersero | 0 | 31 | 0.0 | 0.0-112.2 |  |  |  |
| Haemoctin SDH | 1 | 372 | 2.7 | 0.1-14.9 |  |  |  |
| Hemophil M | 0 | 103 | 0.0 | 0.0-35.2 |  |  |  |
| Immunate | 2 | 972 | 2.1 | 0.2-7.4 |  |  |  |
| Koate DVI | 0 | 17 | 0.0 | 0.0-195.1 |  |  |  |
| Octanate (LV) | 0 | 745 | 0.0 | 0.0-4.9 |  |  |  |

Table S2 Inhibitor development according to concentrate in non-severe hemophilia B

| **Concentrate** | **Inhibitor**  **(N)** | **Treat-ment years**  **(N)** | **inhibitor rate/**  **1000 yrs** | **CI** |
| --- | --- | --- | --- | --- |
| **Hemophilia B** |  |  |  |  |
| Benefix | 1 | 5813 | 0.2 | 0.0-1.0 |
| Rixubis | 0 | 125 | 0.0 | 0.0-29.1 |
|  |  |  |  |  |
| Alprolix | 0 | 770 | 0.0 | 0.0-4.8 |
| Idelvion | 0 | 244 | 0.0 | 0.0-15.0 |
| Refixia | 0 | 120 | 0.0 | 0.0-30.3 |
|  |  |  |  |  |
| Alphanine | 0 | 83 | 0.0 | 0.0-43.5 |
| Berinin | 0 | 164 | 0.0 | 0.0-22.2 |
| BETAFACT | 0 | 460 | 0.0 | 0.0-8.0 |
| Factor IX Grifols | 0 | 50 | 0.0 | 0.0-71.1 |
| Faktor IX SDN (Biotest) | 0 | 1 | 0.0 | 0.0-975.0 |
| Haemonine | 0 | 33 | 0.0 | 0.0-105.8 |
| Immunine | 0 | 615 | 0.0 | 0.0-6.0 |
| Mononine | 0 | 38 | 0.0 | 0.0-92.5 |
| Nanofix | 0 | 44 | 0.0 | 0.0-80.4 |
| Nanotiv | 0 | 45 | 0.0 | 0.0-78.7 |
| Nonafact | 0 | 34 | 0.0 | 0.0-102.8 |
| Octanine | 0 | 447 | 0.0 | 0.0-8.2 |
| Replenine VF | 0 | 152 | 0.0 | 0.0-4.2 |

No concentrates with a minimum of 1000 treatment years available for comparison to inhibitor rate on Benefix
