# Supplementary material for: Inhibitor development in nonsevere hemophilia: data from the European Haemophilia Safety Surveillance (EUHASS) registry
Source: Res Pract Thromb Haemost. 2025 May 17;9(4):102887. doi: 10.1016/j.rpth.2025.102887 (PMC12178916; doi:10.1016/j.rpth.2025.102887)
Supplement: Supplementary Data 2 [file mmc2.docx]

| **Country** | **EUHASS Centre Name** | **Haemophilia Director Surname** | **Director E-mail** |
| --- | --- | --- | --- |
| Austria | Vienna - adults | Cihan Ay | [cihan.ay@meduniwien.ac.at](mailto:cihan.ay@meduniwien.ac.at) |
| Austria | Vienna - children | Christoph Male | christoph.male@meduniwien.ac.at |
| Belgium | Brussels | Cedric Hermans | Cedric.Hermans@uclouvain.be |
| Belgium | Leuven | Peter Verhamme | peter.verhamme@uzleuven.be |
| Bulgaria | Sofia | Toshko Lissitchkov | t_lissitchkov@yahoo.com; t.lissitchkov@abv.bg |
| Cyprus | Nicosia | Marios Antoniades | elemar@cytanet.com.cy |
| Czech republic | Brno - adults | Miroslav Penka | penka.miroslav@fnbrno.cz |
| Czech republic | Brno - children | Jan Blatny | jblatny@icloud.com |
| Czech republic | Prague | Vladimir Komrska | Vladimir.Komrska@fnmotol.cz |
| Denmark | Aarhus | Lone Hvitfeldt Poulsen | Hvitfeldt@ki.au.dk |
| Denmark | Copenhagen | Peter Kampmann | Peter.Kampmann@regionh.dk |
| Finland | Helsinki | Anna-Elina Lehtinen | [anna-elina.lehtinen@hus.fi](mailto:anna-elina.lehtinen@hus.fi) |
| France | Lille | Sophie Susen | [sophie.susen@chu-lille.fr](mailto:sophie.susen@chu-lille.fr) |
| France | Lyon | Yesim Dargaud | [ydargaud@univ-lyon1.fr](mailto:ydargaud@univ-lyon1.fr) |
| France | Montpellier | Christine Biron | c-biron@chu-montpellier.fr |
| France | Paris - Bicetre | Roseline D'Oiron | roseline.doiron@aphp.fr |
| France | Paris - Necker | Annie Harroche | annie.harroche@aphp.fr |
| Germany | Berlin | Robert Klamroth | robert.klamroth@vivantes.de |
| Germany | Bonn | Johannes Oldenburg | johannes.oldenburg@ukb.uni-bonn.de |
| Germany | Bremen | Martina Buehrlen | martina.buehrlen@klinikum-bremen-mitte.de |
| Germany | Frankfurt | Wolfgang Miesbach | Wolfgang.Miesbach@kgu.de |
| Germany | Hamburg-Eppendorf | Florian Langer | langer@uke.de |
| Germany | Munich - adults | Patrick Spannagl | michael.spannagl@med.uni-muenchen.de |
| Germany | Munich - children | Martin Oliveri | [martin.oliveri@med.uni-muenchen.de](mailto:martin.oliveri@med.uni-muenchen.de) |
| Greece | Athens - Aghia Sophia | Helen Platokouki | eplatokouki@paidon-agiasofia.gr |
| Greece | Athens - Hippocration | Efrosyni Nomikou | efrosyni.nomikou@gmail.com |
| Greece | Athens - Laikon | Olga Katsarou | [olgkats@hotmail.com](mailto:olgkats@hotmail.com) |
| Greece | Thessaloniki | Vasileia Garypidou | gali@auth.gr |
| Greece | Thessaloniki - children | Marina Economou | marina@med.auth.gr |
| Hungary | Budapest | Laszlo Nemes | lnemes@t-online.hu |
| Ireland | Dublin - Our Lady's Children's Hospital | Beatrice Nolan | beatrice.nolan@olchc.ie |
| Ireland | Dublin - St. James's Hospital | Niamh O'Connell | [noconnell@stjames.ie](mailto:noconnell@stjames.ie) |
| Italy | Castelfranco Veneto | Radossi Paolo | paolo.radossi@iov.veneto.it |
| Italy | Florence | Giancarlo Castaman | castaman@aou-careggi.toscana.it |
| Italy | Milan | Prof. Flora Peyvandi | flora.peyvandi@unimi.it |
| Italy | Napoli | Angiola Rocino | not responding to email address we had |
| Italy | Padova | Ezio Zanon | ezio.zanon@unipd.it |
| Italy | Parma | Annarita Tagliaferri | atagliaferri@ao.pr.it |
| Italy | Perugia | Giancarlo Agnelli | [giancarlo.agnelli@unipg.it](mailto:giancarlo.agnelli@unipg.it) |
| Italy | Rome - Catholic University | Raimondo De Crisotofaro | Raimondo.Decristofaro@unicatt.it |
| Italy | Turin | RETIRES NOV 2016 Piercarla Schinco | pcschinco@hotmail.com |
| Italy | Vicenza | Alberto Tosetto | alberto.tosetto@aulss8.veneto.it |
| Latvia | Riga | Sandra Lejniece | lejniece@latnet.lv |
| Lithuania | Klaipeda | Neringa Gailiute | neringa.gailiute@gmail.com |
| Malta | Malta | Alexander Gatt | alexander.a.gatt@gov.mt |
| Netherlands | Groningen | Anja Mäkelburg | a.b.u.makelburg@umcg.nl |
| Netherlands | Nijmegen | Britta Laros‐van Gorkom (adults) + Paul Brons (paediatrics) | Britta.Laros-vanGorkom@radboudumc.nl; Paul.Brons@radboudumc.nl |
| Netherlands | Rotterdam | Frank W.G. Leebeek | f.leebeek@erasmusmc.nl |
| Netherlands | Utrecht | Roger Schutgens | r.schutgens@umcutrecht.nl |
| Poland | Warsaw | Jerzy Windyga | jwindyga@ihit.waw.pl |
| Portugal | Lisbon - Hospital de Santa Maria | Cristina Catarino | cristina.criscat@gmail.com |
| Portugal | Lisbon - Hospital de Sao Jose | Anabela Aires | [Anabela.aires@chlc.min-saude.pt](mailto:Anabela.aires@chlc.min-saude.pt) |
| Portugal | Ponta Delgada (Azores) | Cristina Fraga | cristinafragabarros@gmail.com |
| Portugal | Porto - Santo Antonio 2015 | Sara Morais | saratsmorais@hotmail.com |
| Portugal | Porto - Sao Joao | Fernando Araújo |  |
| Romania | Timisoara - External Romania | Margit Serban | mserban@spitalcopiitm.ro |
| Romania | Timisoara - Own patients | Margit Serban | mserban@spitalcopiitm.ro |
| Russia | Samara | Igor Davydkin | dagi2006@rambler.ru |
| Slovakia | Bratislava | Angelika Batorova | batorova@hotmail.sk |
| Slovenia | Ljubljana | Sasa Anzej Doma | [sasa.anzejdoma@gmail.com](mailto:sasa.anzejdoma@gmail.com) |
| Spain | Barcelona | Laura Segura Martinez | lsegura@vhio.net |
| Spain | Malaga | Angeles Palomo Bravo | [anpabra1@yahoo.es](mailto:anpabra1@yahoo.es) |
| Spain | Oviedo | Immaculada Soto Ortega | isotor60@gmail.com |
| Spain | Valencia | Santiago Bonanad | sbonanad@gmail.com |
| Sweden | Gothenborg | Fariba Baghaei | Fariba.Baghaei@vgregion.se |
| Sweden | Malmo | Jan Astermark | Jan.Astermark@med.lu.se |
| Sweden | Stockholm | Margareta Holmström | margareta.holmstrom@karolinska.se |
| Switzerland | Geneva | Pierre Fontana | pierre.fontana@hucge.ch |
| Switzerland | Zurich - children | Markus Schmugge | Markus.Schmugge@kispi.uzh.ch |
| Turkey | Istanbul | Bulent Zulfikar | bulent.zulfikar@istanbulmedicare.com |
| Turkey | Izmir | Kaan Kavakli | kaan.kavakli@ege.edu.tr |
| UK | Aberdeen | Mohammed Khan | [mohammed.khan3@nhs.scot](mailto:mohammed.khan3@nhs.scot) |
| UK | Belfast | Gary Benson | gary.benson@belfasttrust.hscni.net |
| UK | Birmingham - adults | Will Lester | Will.Lester@uhb.nhs.uk |
| UK | Edinburgh | Page Andrew | andrew.page@nhslothian.scot.nhs.uk |
| UK | Glasgow - adults | Bagot Catherine | catherine.bagot@ggc.scot.nhs.uk |
| UK | Glasgow - children | Fernando Pinto | [fernando.pinto@ggc.scot.nhs.uk](mailto:fernando.pinto@ggc.scot.nhs.uk) |
| UK | Leicester | Styliani Salta | [styliani.salta@nhs.net](mailto:styliani.salta@nhs.net) |
| UK | Liverpool - adults | Cathy Farrelly | cathy.farrelly@liverpoolft.nhs.uk |
| UK | London - Great Ormond Street | Mary Matthias | [mary.mathias@gosh.nhs.uk](mailto:mary.mathias@gosh.nhs.uk) |
| UK | London - Hammersmith | Mike Laffan | m.laffan@imperial.ac.uk |
| UK | London - Royal Free | Yee Thynn Thynn | thynn.yee@nhs.net |
| UK | London - Royal London | Vickie Mcdonald | [vickiemcdonald@nhs.net](mailto:vickiemcdonald@nhs.net) |
| UK | London - St. George's | Steve Austin | [steveaustin@nhs.net](mailto:steveaustin@nhs.net) |
| UK | London - St. Thomas' | Madan Bella | Bella.Madan@gstt.nhs.uk |
| UK | Manchester - adults | Charles Hay | [charles.hay@mft.nhs.uk](mailto:charles.hay@mft.nhs.uk) |
| UK | Manchester - children | John Grainger | john.grainger@mft.nhs.uk |
| UK | Newcastle | Kate Talks | kate.talks@nhs.net |
| UK | Oxford | Susie Shapiro | Susie.Shapiro@ouh.nhs.uk |
| UK | Sheffield - adults | Rhona Maclean | rhona.maclean5@nhs.net |
| UK | Sheffield-children | Jeanette Payne | jeanette.payne@sch.nhs.uk |
